# Autonomous nervous system modulation in supine and standing postures in children with probable developmental coordination disorder

**DOI:** 10.1016/j.heliyon.2021.e06111

**Published:** 2021-01-30

**Authors:** Daniel T. Gama, Marcela C. Ferracioli-Gama, José A. Barela, Anielle C.M. Takahashi, Ana Maria Pellegrini, Cynthia Y. Hiraga

**Affiliations:** a Faculty of Education, Federal University of Grande Dourados, Dourados, MS, Brazil; b Department of Physical Education, São Paulo State University, Rio Claro, SP, Brazil; c Institute of Physical Education and Sports, Federal University of Ceará, Fortaleza, CE, Brazil; d Department of Physical Therapy, Federal University of São Carlos, São Paulo, SP, Brazil

**Keywords:** Developmental coordination disorder (DCD), Autonomous nervous system, Heart rate variability, Stress and anxiety

## Abstract

**Background:**

Children with Developmental Coordination Disorder (DCD) are known to have poor physical fitness and psychosocial problems. The autonomous nervous system (ANS) plays an essential role in the regulation of human neurophysiological processes. Inadequate ANS modulation has been associated with harmful health conditions such as poor aerobic power, high body mass index, and symptoms of stress and anxiety. Modulation of ANS in children with DCD needs to be further investigated taking into consideration variables that may influence its function. For instance, would the level of physical fitness or the symptoms of stress and anxiety affect the ANS modulation of children with DCD?

**Aims:**

To examine the ANS modulation during supine and standing postures, and stress/anxiety with questionnaire data from children with probable-DCD (p-DCD).

**Methods:**

and procedures: Thirty children, 8–12 years old, composed two groups paired by age, gender, peak volume of oxygen uptake (aerobic power), and body mass index (BMI): p-DCD (9 boys, mean age 10.8 y) and typically developing (TD). Both groups were compared for stress/anxiety assessment by questionnaire and spectral, symbolic, and complexity heart rate variability (HRV) analyses during posture changes.

**Outcomes and results:**

p-DCD group showed higher stress symptoms than TD group for stress/anxiety assessment in the questionnaire's data, but HRV analyses showed no differences between the two groups. Both groups showed parasympathetic prevalence during supine posture and sympathetic prevalence during standing posture.

**Conclusions and implications:**

Children with p-DCD had similar autonomic control function as TD children during posture change (supine to standing). Symptoms of stress and anxiety demonstrated by p-DCD did not impact their ANS modulation. These results indicate that aerobic power and BMI are probable protective factors of ANS modulation for these children.

## 1 Introduction

Developmental Coordination Disorder (DCD) is identified by difficulties to perform regular fine and/or gross motor tasks, such as writing, cutting, drawing, running, playing games and sports. It affects between 1.8% (Wilson et al., 2017) to 24% (Valentini et al., 2017) of children in school age. Diagnosis criteria for DCD include that the child's motor ability should be well below the expected by age, movement difficulty would negatively impact activities of daily living and/or academic/school productivity, as well as normal neurological and/or intellectual functioning (American Psychiatric Association, 2013). Their motor difficulties are constantly challenging them to deal with bullying and/or exclusion by the classmates to play, misunderstanding on the part of their parents, and teachers on their condition due to laziness or lack of effort (Zwicker et al., 2018). This inappropriate context negatively impacts DCD children regular development in different domains, such as motor, physical, academic, and social-affective and psychosocial (Karras et al., 2019; Leonard, 2016).

A common concern in the literature are the psychosocial problems showed by DCD children. A number of studies (Piek et al., 2008; Pratt and Hill, 2011; Skinner and Piek, 2001) based on observational behavior and standardized questionnaires showed more symptoms of depression (Campbell et al., 2012) and stress/anxiety (Pratt and Hill, 2011) such as sadness, agitation and panic, low self-esteem in children with DCD compared with their typically developing peers. Further, a longitudinal study by Piek et al. (2010) showed that motor difficulties at infancy and early childhood are related with psychosocial symptoms during adolescence. Motor difficulties of children with DCD appear to substantially affect psychosocial behavior from infancy through adolescence. The modulation of ANS, however, remains open for an objective neurophysiological assessment of stress/anxiety in children with DCD (Chalmers et al., 2014).

ANS is composed by the sympathetic (SNS) and parasympathetic (PNS) nervous system, which are also known as the fight-or-flight mechanism and the relaxation response. Stress and anxiety are adaptations of the body in response to a negative experience in preparation for fight-or-flight. The mechanisms of SNS, which trigger stress and anxiety, activate the hypothalamic-pituitary-adrenal axis and promote the secretion of corticotropin-releasing hormone by the hypothalamus. When the corticotropin-releasing hormone reaches the tissue of the adrenal glands, it stimulates the secretion of cortisol, the primary stress hormone (Yvonne and James, 2009). As the plasma cortisol concentration increases in the brain, the neurons receptors of these glucocorticoids become highly occupied and saturated, decreasing their ability to control and inhibit corticotropin through negative feedback in the limbic system (Joëls et al., 2008). Such effects occur when exposure to cortisol is long-lasting and during the state of chronic stress and anxiety, that is, with predominant SNS modulation.

In recent years, there has been an increase in the number of studies using Heart rate variability (HRV) as an accepted biomarker for ANS modulation and stress and anxiety (Kim et al., 2018). Technically, HRV is the variation in consecutive heartbeats over time (Acharya et al., 2006). The periods of time between successive heart beats are known as RR intervals which depend on the extrinsic regulation of the heart rate (HR). HRV reflects the heart's ability to adapt to circumstances by detecting and quickly responding to stimuli. Recently, Shaffer and Ginsberg (2017) argued that HRV measurements also assess the neurocardiac function generated by heart-brain interactions as well as dynamic non-linear ANS processes.

The balancing activity of the SNS and PNS divisions of the ANS control the heartbeats. Increased SNS or reduced PNS activity results in heartbeat acceleration (Acharya et al., 2006). In contrast, a low SNS activity or a high PNS activity cause heartbeat deceleration. According to Lipsitz and Goldenberg (1992) and Peng et al. (1994), an organism's optimal adaptation to internal and environmental demands is achieved by this variability function in heartbeat. Moreover, adaptability variability patterns of an organism can be determined by different factors, such as genetic, environmental, and clinical conditions. On the contrary, when the ANS is not adapted to load demands, the organism becomes more vulnerable to disease. Low HRV is frequently associated with ANS imbalances, maladaptation, and disease, including stress and anxiety (Chalmers et al., 2014; Peng et al., 1994).

Few studies focused on investigating cardiac function in DCD (Cavalcante Neto et al., 2018; Coverdale et al., 2012). Recently, Cavalcante Neto et al. (2018) compared the HRV in supine and standing postures in children with DCD and typically developing children. Results showed that children with DCD presented higher sympathetic and lower parasympathetic activity in the supine position. That is, children with DCD exhibit lower complexity and adaptability of ANS control when the body is in resting position compared to TD children. On the other hand, Coverdale et al. (2012) showed results with no significant differences in HRV linear components between TD group and p-DCD in supine position. Moreover, they argue that children with DCD still show low parasympathetic modulation and a certain imbalance in the sympathovagal relationship in supine position based on the differences between groups on baroreflex sensitivity measure. The authors explain that this result was due to higher body fat percentage in the p-DCD compared to the TD group.

While Coverdale et al. (2012) argued the effect of low BRS in adolescents with p-DCD occurred as a result of high body fat percentage rather than motor difficulties itself, Cavalcante Neto et al. (2018) argued that lower parasympathetic and higher sympathetic modulations in supine position in children with DCD compared with their typical developing children peers were only due to motor deficits. A relevant aspect in Cavalcante Neto et al. (2018) was that there were no differences in mean age, BMI, and level of physical activity between the DCD and typically developing groups.

Modulation of ANS in children with DCD needs to be further investigated taking into consideration variables that may play a role in the cardiac function. For instance, would the level of physical fitness affect the ANS modulation of children with DCD? Children with DCD frequently show low levels of physical fitness probably because of their motor deficits (Hiraga et al., 2014). Motor deficits per se might not be a candidate for the low ANS modulation of DCD. Other associated variables that directly influence the ANS modulation may be causing such alterations on children with DCD. It is well established in the literature that variables such as gender, age, aerobic power (Michels et al., 2013), and BMI (Altuncu et al., 2012; Birch et al., 2012; Gutin et al., 2000; Paschoal et al., 2009) influence autonomic modulations. Our study aims to (i) examine the ANS modulation during supine and standing postures in children with DCD, and (ii) to examine the stress and anxiety symptoms with a questionnaire applied to children with DCD.

## 2 Method

### 2.1 Participants

A total of 198 children from two public schools, aged eight to 12 years, were initially invited to take part in the study. A group of 88 children [50 girls (mean = 10.2 years); 38 boys (mean = 10.3 years)] provided a written consent by their parents or legal guardian for participating in the study. These children also voluntarily assented to participate in each experimental phase of the study. According to school's records, children participating in this study had no neurological problems and/or intelligence deficit, as per routine screening test by specialists. All study procedures were approved by the Ethics and Research Committee of the Sao Paulo State University.

### 2.2 Procedures

#### 2.2.1 Motor coordination assessment (MABC-2)

Motor coordination of the 88 children was assessed using the Movement Assessment Battery for Children-2 (MABC-2, Henderson et al., 2007). The assessment was performed at the children's schools in an appropriate room prepared for this purpose. Each child was evaluated individually and the whole assessment took between 30 to 40 min per child. According to MABC-2 instructions, 21 children (13 male) were classified with significant movement difficulty (below the 5^th^ percentile), 16 children (one male) were at risk of having movement difficulty (between the 5^th^ and 15^th^ centile), and 51 children (24 males) with no movement difficulties (above the 15^th^ centile).

#### 2.2.2 Body mass index (BMI) and aerobic power (VO_2 peak_) assessment

Following the MABC-2 assessment, anthropometric measures were taken from and aerobic power tests were performed in each child on the second day of the study. These sessions occurred in groups with a maximum of five children with a duration of about 20 min per group. Weight and height were measured using a digital scale and a stadiometer, respectively. The median of three measures for weight and height was used to calculate the body mass index (BMI) of each child.

The aerobic power (VO_*2 peak*_) test was the maximum multi-stages 20 m Shuttle Run – 20-MST (Leger and Lambert, 1982). Considering the movement difficulty of children participating in the study, this test was adapted with the presence of a research collaborator as a guide who helped children with the running speed of 20-MST stages. The need to use the guide became evident after preliminary data collection, in which children showed difficulties to match their running speed to the pace dictated by a record emitting tones at appropriate intervals.

Prior to the beginning of the 20-MST test, the children were instructed to run side by side with the guide as long as possible (maximum of their capability). The test was interrupted when the children failed twice consecutively to reach the spatial and temporal matching goals required, getting delayed in relation to the audio.

#### 2.2.3 Stress/anxiety assessment – Children's stress scale (CSS)

On the third day of experimental procedures, the children answered the Children Stress Scale (CSS) (Lipp and Lucarelli, 1998). This scale was validated for children from six to 14 years old (Lucarelli and Lipp, 1999), and it identifies the symptomatology of stress/anxiety, discriminating physical and psychological reactions. The CSS consists of 35 items placed on a Likert scale from zero to four points. The higher the score on the scale, the greater the stress reactions. The score of the CSS is grouped into four reactions factors: Physical Reactions (PhR), Psychological Reactions (PsR), Psychological Reactions with Depressive Component (PRDC), and Psychophysiological Reactions (PPR). The CSS was applied by an accredited psychologist in a private room of the children's school.

#### 2.2.4 Grouping matched by gender, age, BMI and VO_2 peak_ outcomes

Two groups were selected for the HRV measure procedures. Sixteen children scoring ≤5th centile on the MABC-2 composed the probable DCD (p-DCD) group while 16 other children matched for gender, age, BMI, VO2 outcome, and scoring >15th centile composed the typically developing (TD) group. The procedure did not consider all criteria described in the Diagnostic and Statistical Manual of Mental Disorders for DCD (American Psychiatric Association, 2013). We were not able to determine, for instance, whether children's motor difficulties were affecting their activities of daily living (Criterion B). Therefore, the term p-DCD was adopted. Following Smits-Engelsman et al. (2015), children identified with severe movement difficulty, but with one or more criteria for DCD not evaluated, were reported as with p-DCD.

From 21 children identified with p-DCD based on the MABC-2, five were unable to participate in the study. Three of them with extremely high BMI and/or extremely low aerobic power. Two children were not allowed by their parents to continue to participate on the experimental HRV procedure. One child with p-DCD was excluded due to the artifact on the electrocardiogram raw data collected for the further HRV analyses. Thus, p-DCD and TD groups were formed by 15 children (nine boys and six girls). Girls in both groups were asked about menarche and answered that they had not reached it at the time of HRV data collection.

Table 1 presents the matching of the p-DCD and TD groups. A Student's T-Test was applied to compare the two groups' Age, BMI, VO_2__peak_, and MABC-2 by component and total standard score. The groups did not show significant differences regarding age, BMI and VO_2__peak_. Differences on the MABC-2 components and total scores were statistically significant between the groups.

**Table 1:** Groups matching relate to age, Body Mass Index (BMI), aerobic power (VO2 peak) and MABC-2 performance.

	p-DCD	TD	*P*
Boy (n)	9	9	
Girl (n)	6	6	
Age (months)	129.7 (14.8)	126.0 (14.6)	NS
BMI (kg/m^2^)	21.6 (4.3)	19.8 (4.0)	NS
Aerobic power (VO_2_ peak)	44.2 (3.7)	45.0 (3.7)	NS
MD Score – MABC-2	6.1 (1.7)	10.0 (1.5)	<0.01
BS Score – MABC-2	6.7 (2.1)	11.0 (2.5)	<0.01
BA Score – MABC-2	3.6 (1.7)	8.8 (2.1)	<0.01
Total score – MABC-2	4.0 (1.3)	9.8 (1.5)	<0.01
Percentile TOTAL	3.1 (2.1)	47.6 (19.1)	<0.01

MD – Manual Dexterity; BS – Ball Skills; BA – Balance; DCD - Developmental Coordination Disorder; TD – Typical Developing; P – p-value on Student's T-Test (significance level p < .05); NS = non-significant.

#### 2.2.5 RR interval data in supine and standing up postures

RR interval data collection for Heart Rate Variability analysis was conducted in the laboratory at the university in two sessions. In the first session, participants visited the laboratory for the purpose of adapting to the environment prior to the experimental procedures for data collection. They were familiarized with the data collection equipment and had their blood pressure recorded. For the RR interval data collection, a Polar heart rate monitor (RS800 CX model), an elastic band with same-brand receiver, and a transmitter, all validated for children, were used (Gamelin et al., 2008). At the end of the visit, a date was scheduled for the sequence of the experimental protocol. On the day before and at the day of experimental data collection, participants and their parents or legal guardians were instructed to: (a) not perform vigorous physical activity; (b) not consume stimulating beverages or foods (e.g., coffee, cola, energy drinks, chocolates, energy bars); and (c) have a good night of sleep.

The second session for RR interval data collection was always scheduled in the morning between 8:00 am and 10:00 am to minimize differences from the circadian rhythm. This procedure was to control the variation of the ambient conditions (temperature and relative humidity), ensuring favorable environmental conditions for RR interval data collection. At all stages of data collection, the chronometer was synchronized with the heart rate monitor. The respiratory rate per minute was observed by the participants' chest movement and recorded by a collaborating researcher. This procedure allowed examining the occurrence of respiration synchronization with parasympathetic autonomic nervous system activity by modulation. Only data with respiratory rate between 12 and 25 per minute were considered for further analyses.

On the second session, the experimenter checked if the participant followed the previous guidelines for data collection. If not, the child was asked to return another day. If so, the resting heart rate and blood pressure of each child were measured. If heart rate and blood pressure were normalized at rest according to the reference recorded at the previous session, the participant was considered fit to proceed with the experimental protocol. If heart rate or blood pressure were altered at rest with changes above 15% compared to the previous visit, the participant was instructed to return another day. For the children considered fit, the elastic bands with heart rate receiver were placed on the thoracic waist at the children's xiphoid process and its data synchronization with the heart rate monitor was tested. The relative humidity and ambient temperature were measured and recorded with a hygro thermometer. Relative humidity above 40% and below 70% and ambient temperatures between 20° and 24 °C were considered adequate for data collection. Otherwise, data collection was canceled and scheduled for another day.

After fulfilling the above prerequisites, the children were instructed to remain quiet, to not talk nor fall asleep during the data collection procedures. The children were also asked to lay down on the stretcher supine for 10 min to stabilize their heart rate at this position. After heart rate stabilization, the researcher synchronized a stopwatch with the monitor clock to begin collecting RR interval data in the supine position for 8 min. Then, the children were required to stand up and remain in the standing and quiet position for another 8 min for RR interval data collection.

### 2.3 Data analysis

The RR interval data collected from the heart rate monitor were transferred to a computer for data processing. Data were extracted in text format from the Pro-trainer 5 software (Polar Electro Oy) and tabulated.

The heart rate autonomic modulation was evaluated by heart rate variability (HRV) - linear and nonlinear parameters. For the analyzes, 256 RR interval sequences with the greatest stability were chosen for supine and standing positions (TASK FORCE, 1996). Evident non-stationary series, as well as progressive increases or decreases or sudden variance changes were excluded (Magagnin et al., 2011). No filter or manual substitution of RR intervals were performed in a presence of artifacts in tachogram. In case of presence of artifacts in 256 RR interval sequences, another sequence was chosen and if it was not possible, the participant was excluded.

The spectral analysis of the RR interval data time series (256 points) was performed using the autoregressive model algorithms (Malliani and Montano, 2002; Pagani et al., 1986) (software AR24.exe version 1.2). Then, the area of low frequency (LF – between 0.04 and 0.15 Hz) and high frequency (HF – between 0.15 and 0.40 Hz) components of the HRV were calculated for further analyses. The LF and HF bands that best represent the sympathetic and vagal cardiac modulation, respectively, were used for this study (TASK FORCE, 1996). Linear components of the HRV were expressed as normalized units (LFnu) and absolute units (HFabs), the LFabs/HFabs ratio (LF/HF) (between low frequency and high frequency bands) indicating sympathovagal balance was also included for data analyses (Lipsitz and Goldberger, 1992). Normalization of LF was performed by dividing LFabs by total power (VLFabs + LFabs + HFabs) and by multiplying the ratio by 100 (Malliani and Montano, 2002; Pagani et al., 1986).

Recently, some possible biased proxies in RR interval indices have been pointed by researchers (Malik et al., 2019; Boyett et al., 2019), mainly related to the nonlinear relationship between the RR interval mean and the magnitude of changes in RR intervals. Following La Rovere et al. (2020), the interpretation of RR interval comparisons proxies is preserved as long as groups and experimental conditions show the same RR interval mean. In the case of variation among groups and conditions of RR interval mean, LF/HF ratio should be checked for potential variations before interpretation of HRV indices expressed in absolute units. Alternatively, it is suggested to use RR interval indices that feature an intrinsic normalization, such as normalized units or LF/HF ratio.

Nonlinear parameters are well accepted for HRV analysis (Guzzetti et al., 2005; Porta et al., 2007a). HRV calculations of RR interval data time series (256 points) were performed with symbolic analysis (Porta et al., 2001) and conditional entropy (Porta et al., 1998). Studies involving pharmacological blockades or autonomic tests showed that the Zero Variation (0V%) symbolic index is related to cardiac sympathetic modulation, and Two Unlike Variation (2UV%) symbolic indexes are related to vagal modulation (Guzzetti et al., 2005; Porta et al., 2007b). The conditional entropy (CE) is a complexity measure used to quantify the amount of information carried by the most recent sample of patterns that cannot be derived from a sequence of values of the length of past patterns. The CE was assessed by the normalized complexity index (NCI), which expresses the complexity in terms of dimensionless units, this index ranges from 0 (null information) to 1 (maximum information). The larger both indexes are, the greater the complexity and the lower the regularity (Porta et al., 2007a).

### 2.4 Statistical analyses

Statistical analyses were performed using Statistica 7.0 for Windows. Each data set obtained from the CSS (mean of each Reaction Factor and sum of Factors) and HRV indices (mean RR interval, LF un, HF abs, LF/HF, 0V%, 2UV% and NCI) were tested separately for normality with the Shapiro Wilk's W test. Data that met normality were analyzed with parametric statistics while data that did not meet normality were analyzed with nonparametric statistics. Tests for gender were performed for each of the variables above and no differences were found. Thus the children were grouped as p-DCD and TD, regardless of gender. For parametric statistics, independent Student T-Test and paired Student T-Test were applied for intergroup and intragroup comparisons, respectively. Cohen's *d* effect sizes calculations were applied to measure significant differences' magnitudes, with range for small = 0.2, medium = 0.5, and large = 0.8 (Cohen, 2013). For nonparametric statistics, Mann-Whitney U and Wilcoxon paired test were applied for intergroup and intragroup comparisons, respectively. Effect sizes *r* calculations were applied to measure significant differences' magnitudes, with range for small = 0.1, medium = 0.3, and large = 0.5 (Rosenthal, 1994).

## 3 Results

### 3.1 Intergroup comparisons for HRV in supine and standing postures

#### 3.1.1 Linear components

**Figure 1:**
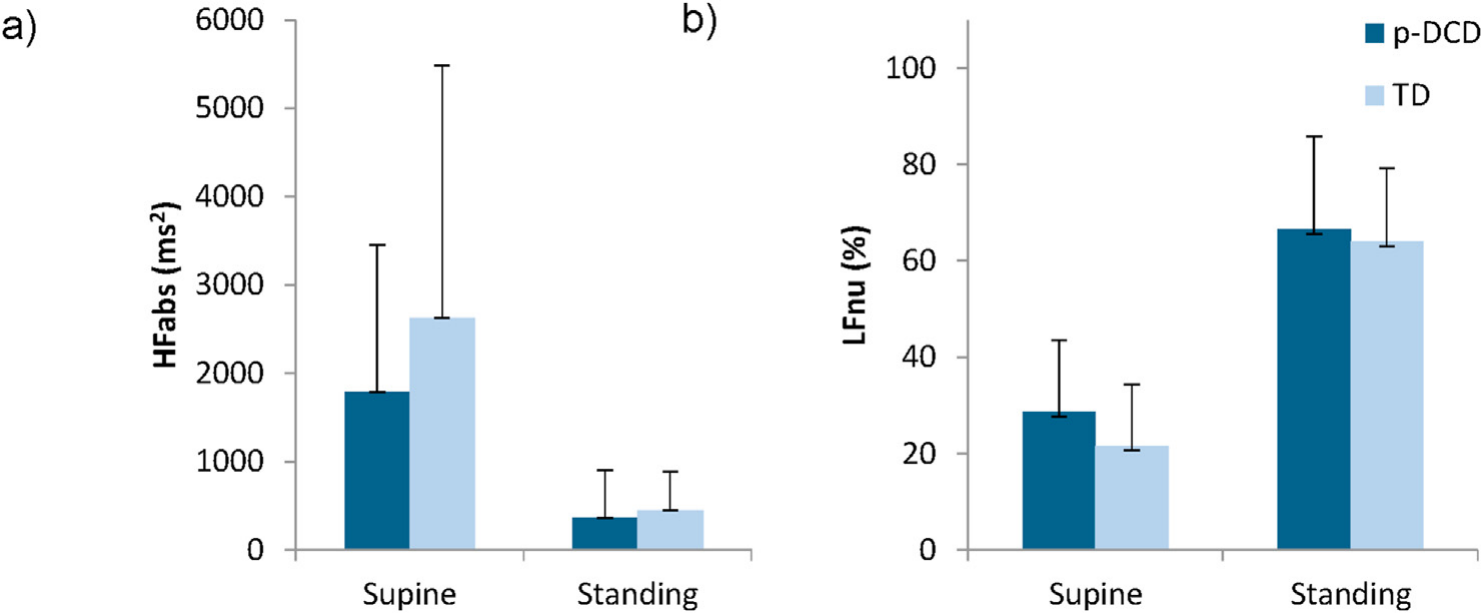
Mean (standard deviation) for HFabs (1a) and LFun (1b) spectral components of Heart Rate Variability in supine and standing postures for p-DCD and TD groups. *HFabs – High Frequency in absolute values; LFun – Low Frequency in normalized units; p-DCD – probable developmental coordination disorder; TD – typically developing*.

Figure 1 shows the HFabs (1a) and LFnu (1b) values of the HRV spectral analysis in supine and standing stances.

The results of the Students' T and Mann-Whitney's U tests did not reveal any significant difference between the p-DCD and TD groups for LFun in supine [t (28) = 1.41 p = 0.16] and standing [t (28) = 0.39 p = 0.69], HFabs in supine [Z = 0.68 p = 0.49] and standing [Z = 1.05 p = < 0.29], RR interval in supine [t (28) = 1.80 p = 0.08] and standing [t (28) = 1.70. p < 0.09], and LF/HF in supine [Z = 0.91 p = 0.36] and standing [Z = 0.72 p = 0.46].

#### 3.1.2 Nonlinear components

Consistent with the results of the linear HRV analyses, results of the T-tests on the nonlinear variables did not indicate any difference between the p-DCD and TD groups for 0V% in supine [t (28) = 1.20 p = 0.23] and standing [t (28) = 0.33 p = 0.73], 2UV% in supine [t (28) = 1.35 p = 0.18] and standing [t (28) = 1.44 p = 0.16], and NCI in supine [t (28) = 0.46 p = 0.64] and standing [t (28) = 1.35, p = 0.18].

### 3.2 Intragroup comparisons for HRV in supine and standing postures

#### 3.2.1 Linear components

Table 2 presents the results of the linear intragroup HRV analyses in the supine and standing positions.

**Table 2:** Mean standard deviation for linear components of HRV in supine and standing postures for children with p-DCD and TD.

Linear Analysis	p-DCD				TD			
	Supine	Standing	*P*	*ES*	Supine	Standing	*P*	*ES*
Mean RR-i (ms)	723 (89)	595 (55)	<.001	d = 2.01	786 (101)	630 (59)	<.001	d = 1.93
LFnu (%)	28.6 (14.9)	66.5 (19.3)	<.001	d = 2.13	21.6 (12.8)	64 (15.2)	<.001	d = 2.02
HFabs (ms^2^)	1785 (1670)	365 (535)	< .001^1^	r = 0.62	2630 (2854)	444 (475)	< .01^1^	r = 0.58
LF/HF	0.58 (0.64)	6.61 (13.06)	< .001^1^	r = 0.61	0.33 (0.22)	2.8 (2.26)	< .01^1^	r = 0.61

RR-i – RR interval; Var – Variance of iR-R; LFnu – Low Frequency normalized; HFabs – High Frequency in absolute units, LF/HF – relation between LF and HF; HRV – heart rate variability; p-DCD – probable developmental coordination disorder; TD – typically developing; P – p-value of statistical analyses; ^1^ – non parametric data analyses, ES = Effect Size.

Both groups of children responded to changes from supine to standing stances with statistically significant differences and large effect size. The two groups decreased RR interval values, p-DCD [t (14) = 7.77 p < 0.001] and TD [t (14) = 7.48 p < 0.001], increased LFnu, p-TDC [t (14) = - 8.28 p < 0.001] and TD [t (14) = - 7.84 p < 0.001], decreased HFabs, p-DCD [Z = 3.40 p < 0.001] and TD [Z = 3.23 p = 0.01], and decreased LF/HF, p-DCD [Z = 3.35 p < 0.001] and TD [Z = 3.35 p = 0.01].

#### 3.2.2 Nonlinear components

Table 3 summarizes the results of the non-linear intragroup HRV analyses in supine and standing postures.

**Table 3:** Mean (standard deviation) for nonlinear components of HRV in supine and standing postures for children with p-DCD and TD.

Nonlinear Analysis	p-DCD				TD			
	Supine	Standing	*P*	*ES*	Supine	Standing	*P*	*ES*
0V (%)	11.92 (7.33)	37.07 (11.47)	<0.001	d = 2.47	8.86 (6.56)	35.58 (12.51)	<.001	d = 1.84
2UV (%)	22.22 (6.57)	7.03 (2.92)	<0.001	d = 2.38	25.86 (8.02)	8.94 (4.21)	<.001	d = 2.27
NCI	0.8 (0.05)	0.59 (0.07)	<0.001	d = 2.57	0.81 (0.06)	0.63 (0.09)	<.001	d = 1.62

0V – zero variations in symbolic analysis, 2UV – 2 Unlike Variations in symbolic analysis, NCI – Normalized Complexity Index; HRV – heart rate variability; p-DCD – probable developmental coordination disorder; TD – typically developing; P – p-value of statistical analyses; ES = Effect Size.

Results of the HRV symbolic analyses revealed that both groups responded to postural changes from supine to standing with statistically significant differences and large effect size. The two groups increased 0V, p-DCD Group [t (14) = - 9.59 p < 0.001] and TD Group [t (14) = - 7.15 p < 0.001], decreased 2UV, p-DCD Group [t (14) = 9.25 p < 0.001] and TD Group [t (14) = 8.81 p < 0.001]; and decreased the NCI, p-DCD Group [t (14) = 9.97 p < 0.001] and TD group [t (14) = 6.28 p < 0.001].

### 3.3 Stress and anxiety assessment with questionnaire data

Table 4 presents the results of the Child Stress Scale (CSS) questionnaire.

**Table 4:** Mean (standard deviation) for the stress level for children with p-DCD and TD by the Child Stress Scale (CSS).

Reaction Factor	p-DCD – pts(*s*)	TD – pts*(s)*	*P*	*ES*
*PhR*	13.1 (*8.3*)	6.9 (*7.3*)	0.04	d = 0.79
*PsR*	13.4 (*7.6*)	8.5 (*6.9*)	0.07	d = 0.67
*PsRDC*	11.2 (*7.1*)	5.3 (*5.1*)	0.01	d = 0.95
*PsPhR*	11.6 (*5.9*)	8.33 (*6.5*)	0.16	d = 0.52
SF	49.4 (*26.6*)	29.1 (*21.6*)	0.03	d = 0.72

PhR – Physical Reactions; PsR – psychological reactions; PsRDC – Psychological reactions with depressive component; PsPhR – Psychophysiological reactions; pts(s) - points; SF – sum of factors; p-DCD – probable developmental coordination disorder; TD – typically developing; P – p-value of statistical analyses; ES = Effect Size.

Results from the T-tests revealed statically significant differences and moderate/large effect sizes between groups for Physical Reactions (PhR) [t (28) = - 2.16 p < 0.05], Psychological Reactions with Depressive Component (PsRDC) [t (28) = - 2.61 p < 0.05], and for the sum of the factors (SF) [t (28) = - 2.28 p < 0.05]. In all analyses, the p-DCD group showed higher stress reactions when compared to the TD group.

## 4 Discussion

This study examined the ANS modulation in supine and standing postures in children with p-DCD and TD. The autonomic modulation function is commonly assessed using a protocol of posture changes (i.e., from supine to standing). Healthy individuals, regardless of age, respond with changes in ANS by adapting to active posture change, from a predominant parasympathetic modulation in the supine to a predominantly sympathetic modulation in the standing posture (Alehan et al., 1996; Lewis et al., 1997; Piccirillo et al., 1997; TASK FORCE, 1996). The strength of our study is on the matching strategy of control variables between the two groups. Specifically, BMI and VO_2_ peak performance were controlled to distinguish the possible effect of overweight or obesity and poor cardiorespiratory function on HRV modulation.

Considering the possible biased proxies in RR interval indices for ANS modulation (Malik et al., 2019), in our study, attempting to diminish this possible bias, the interpretation of the results regarding the predominance of sympathetic and parasympathetic modulation between groups and experimental conditions were performed based on an integrated analysis of the linear and nonlinear parameters used. In our study the results obtained in spectral analysis, symbolic, and conditional entropy converges to the same direction. The results of our study indicated both groups showed predominantly parasympathetic modulation in supine posture and sympathetic modulation at standing posture, which is expected for healthy individuals (TASK FORCE, 1996).

Similar to our study's results, Coverdale et al. (2012) showed no differences in the linear HRV components between participants with p-DCD and TD peers. The authors argue that although there were no significant differences between groups, the results of HRV associated with lower HF and Total Power as well as higher LF/HF ratio for p-DCD suggest a decrease in parasympathetic and increase in sympathetic activity. Moreover, Coverdale et al. (2012) showed that baroreflex sensitivity (BPS) was reduced in adolescents with p-DCD mainly due to increased percentage of body fat. BRS is a measure of short-term blood pressure regulation and takes into account alterations in heart rate. Diminished BRS indicates possible cardiovascular morbidity and mortality. Therefore, the diminished baroreflex sensitivity reflects a low parasympathetic modulation and some level of sympathovagal imbalance.

Different to the results of our study, Cavalcante Neto et al. (2018) showed significant differences in linear and nonlinear HRV components between the DCD and TD groups. Their results indicate that children with DCD show a higher predominance of sympathetic modulation in the supine position and therefore lower adaptability to the standing posture change.

A relevant difference between our study and that of Cavalcante Neto et al. (2018) is on the strategy to match participants from both groups. Cavalcante et al. paired children by age, BMI, and levels of physical activity following the Brazilian version of the Physical Activity Questionnaire for Children (PAQ-C). In our study, we adopted a more rigorous matching strategy involving gender, age, BMI, and cardiorespiratory function based on VO_2_ peak measure. Levels of physical activity by PAQ-C may not accurately reflect children's aerobic capacity. Instead, the similar VO_2_ peak measures for both groups in our study may explain the lack of difference between p-DCD and TD for the predominantly parasympathetic modulation in supine and sympathetic at standing posture. It is also relevant to emphasize the control of match pairing by gender, age, BMI, in addition to VO2 max.

To control for certain observable characteristics is necessary to examine the HRV components. According to Michels et al. (2013), HRV is influenced by age, gender, and aerobic capacity. Other authors also describe HRV changes as a function of BMI (Altuncu et al., 2012; Birch et al., 2012; Gutin et al., 2000; Paschoal et al., 2009). Several studies have shown that children with DCD have poor aerobic power and high BMI when compared to TD children (Cairney et al., 2005; Cairney et al., 2010; Wu et al., 2011; Wu et al., 2010). Therefore, studies using HRV component analysis in children should consider controlling for age, gender, aerobic power, and BMI.

In line with results from Cavalcante et al. (2018), Chen et al. (2015) also examined HRV in children with DCD during cognitive tasks at different levels of difficulty. The authors found higher cardiac sympathetic modulation in children with DCD in comparison to TD. In addition, the authors reported a blunted cardiac autonomic adjustment to some cognitive tasks in the DCD compared to the TD group. The authors suggested that the higher cardiac sympathetic modulation in children with DCD might be due to lower levels of aerobic fitness, in agreement with previous findings. Our results ruled out this explanation, since aerobic fitness was similar and to a satisfactory level in both groups.

Regarding to psychosocial problems showed by children with DCD, Cairney et al. (2013) emphasize the need for more research on the causal association between DCD and stress and anxiety symptoms. For instance, research with monozygotic twins (Piek et al., 2007; Pearsall-Jones et al., 2011) has argued that differences between the co-twins would only be explained by environmental factors with no predetermined genetically effects. These studies have found increased depressive and stress and anxiety symptoms in DCD children compared to their typically developed co-twins (Piek et al., 2007; Pearsall-Jones et al., 2011), which support explanations for these problems being mostly due to environmental factors. Longitudinal studies in children with DCD showed a relationship between motor difficulties at an early age and later stress and anxiety symptoms, even if these symptoms were not found at an early age (Piek et al., 2010). These results also provide support and evidence for psychosocial problems in children with DCD being mostly related to environmental factors. Even though these studies used standard research methods, their focus on the etiology factors of psychosocial problems in DCD children does not fully explain the influence of biological factors, since they don't use biological data in their analyses.

The results we obtained with the CSS questionnaire confirmed previous results showing that children with p-DCD have more symptoms of stress and anxiety than TD. Although it is important to verify the symptoms of stress and anxiety in children with DCD with questionnaire data, evaluations in the neurophysiological dimension may also be relevant to understand the impact of these symptoms. To achieve these objectives, evaluations of ANS modulation were performed. The results showed that children with p-DCD did not present different patterns of the ANS modulation function when compared to TD children. Specifically, children with p-DCD did not show neurophysiological symptomatology of stress and anxiety in HRV analysis.

Our results combined with the literature suggest that the neurophysiological system of children with DCD adequately respond and adapt to basic postural demands. Similar to the literature (Cairney et al., 2013; Omer et al., 2019), we showed that even though children with p-DCD presented symptoms of stress and anxiety in the questionnaire assessment, these identified symptoms did not impact their autonomic control. These results indicate that the identified psychosocial problems in p-DCD children are probably not characterized by a biological predisposition, reinforcing the explanations that these difficulties may arise mostly due to environmental factors or else when they are task demanded. In fact, Chen et al. (2015) presents strong evidence that, when compared to a control group and performing a cognitive task with high perception demand, DCD children (9–10 years) have altered and decreased HRV responses.

Despite the important results of our study, some limitations need to be acknowledged. First, from the total population of 198 eligible children meeting the age criterion that were invited to the study, only 88 were authorized by their parents or legal guardians to participate, which may add bias to our sample. The modest sample size is considered a limitation to the generalizability of the study results. Another limitation relates to the fact that only the age group of 8–12 years old was studied. Therefore, analyses in older populations under the same experimental conditions are necessary to infer how the changes in the autonomic control function develops in the DCD population. Another aspect to consider is that just one short-term HRV analysis was performed. To better understand the autonomic control function in DCD children, it would be important to relate HRV analyses with a long-term assessment, potential changes in autonomic control function during motor tasks performance, and during intervention programs.

## 5 Conclusion

This paper adds a new strategy to match groups and examine ANS modulation in supine and standing positions, in which the experimental (children with p-DCD) and control (TD children) group show similar age, gender, aerobic power, and BMI characteristics. Results show that the ANS modulation of children with p-DCD does not differ from TD, and children with p-DCD show healthy adaptation of ANS modulation in supine and standing postures. Symptoms of stress and anxiety demonstrated by children with p-DCD did not impact their ANS modulation. Further, these results indicate that aerobic power and BMI are probable protective factors of ANS modulation for these children.

## Declarations

### Author contribution statement

Daniel T. Gama: Conceived and designed the experiments; Performed the experiments; Analyzed and interpreted the data; Contributed reagents, materials, analysis tools or data; Wrote the paper.

Marcela Ferracioli-Gama, Anielle C. M. Takahashi, Ana Maria Pellegrini, Cynthia Y. Hiraga: Conceived and designed the experiments; Analyzed and interpreted the data; Contributed reagents, materials, analysis tools or data; Wrote the paper.

José A. Barela: Analyzed and interpreted the data; Contributed reagents, materials, analysis tools or data; Wrote the paper.

### Funding statement

This research did not receive any specific grant from funding agencies in the public, commercial, or not-for-profit sectors.

### Data availability statement

The authors do not have permission to share data.

### Declaration of interests statement

The authors declare no conflict of interest.

### Additional information

No additional information is available for this paper.
